# Diagnostic value of salivary CRP and IL-6 in patients undergoing anti-TNF-alpha therapy for rheumatic disease

**DOI:** 10.1007/s10787-018-0515-8

**Published:** 2018-07-24

**Authors:** Dorota Sikorska, Zofia Orzechowska, Rafał Rutkowski, Anna Prymas, Marta Mrall-Wechta, Daria Bednarek-Hatlińska, Magdalena Roszak, Anna Surdacka, Włodzimierz Samborski, Janusz Witowski

**Affiliations:** 1 Department of Rheumatology and Rehabilitation, Poznan University of Medical Sciences, 28-Czerwca 1956 Street 135/147, 61-545 Poznan, Poland; 2 Department of Pathophysiology, Poznan University of Medical Sciences, Poznan, Poland; 3 Department of Conservative Dentistry and Endodontics, Poznan University of Medical Sciences, Poznan, Poland; 4 Department of Computer Science and Statistics, Poznan University of Medical Sciences, Poznan, Poland

**Keywords:** Saliva, C-reactive protein, Interleukin-6, Rheumatic disease, Biologic therapy

## Abstract

**Introduction:**

Saliva has been increasingly used as a diagnostic medium for disease detection and monitoring. The aim of this observational, prospective, pilot study was to investigate whether salivary concentrations of CRP and IL-6 correlate with those in serum and with the clinical course of a rheumatic disease.

**Materials and methods:**

Nineteen patients with rheumatic disease newly scheduled for anti-TNFα therapy were included. Patients received anti-TNFα treatment (adalimumab, certolizumab, golimumab or infliximab) as per standard protocols. CRP and IL-6 were measured with high-sensitivity immunoassays before and after 12 weeks of therapy, according to standard regimens. The data were analyzed with nonparametric statistics.

**Results:**

Concentrations of CRP in saliva correlated significantly with those in serum (*R* = 0.62; *p* < 0.0001) and decreased markedly after successful response to treatment. In patients with a limited response to treatment salivary CRP levels increased. In contrast to CRP, the salivary concentrations of IL-6 did not change significantly over the course of therapy and they did not correlate with serum IL-6 concentrations. Salivary levels of neither CRP nor IL-6 corresponded to parameters of oral health and hygiene.

**Conclusions:**

Salivary CRP but not IL-6 could be of potential use for monitoring the rheumatic disease activity.

## Introduction

Composition of human saliva depends both on systemic and local factors (Khan et al. 2017). Thus, it may reflect pathophysiological processes relevant to the development both of systemic and local disease (Abdul Rehman et al. 2017; Buczko et al. 2015; Cruz-Almeida et al. 2017). In this respect, saliva has been used as a diagnostic tool to aid the assessment of periodontal disease activity (Korte and Kinney 2016; Podzimek et al. 2016). Moreover, saliva gains an increasing attention as a potential analytical medium for endocrinology, neurology, oncology, and infectious diseases (Clements 2013; Khan et al. 2017; Tabak 2007; Wei and Wong 2012). Identification of disease biomarkers in saliva opens a new avenue for screening populations for disease risk and progression as well as response to treatment (Prasad et al. 2016).

The ease and low cost of saliva collection can make it an ideal diagnostic fluid (Prasad et al. 2016). Thus, the obvious question is how well changes in the saliva reflect the course of a disease.

Several mediators of inflammation, collagen breakdown, and/or bone remodeling change clearly in serum of patients with rheumatic diseases (Buczko et al. 2015; Mirrielees et al. 2010). Since saliva contains many of these serum-derived mediators (Buczko et al. 2015; Khan et al. 2017; Mirrielees et al. 2010), they may be of potential use for the rheumatic disease monitoring. Introduction of anti-tumor necrosis factor alpha (anti-TNFα) therapy for rheumatic disease has revolutionized disease control and greatly improved the outcome of rheumatic disease (Mewar and Wilson 2011). Monitoring of a response to therapy involves the measurement of C-reactive protein (CRP) (Gavrila et al. 2016). Typically, a successful treatment is associated with a suppression of serum CRP levels (Buch et al. 2005).

Thus, we have chosen patients with inflammatory arthritis undergoing therapy with anti-TNFα agents to analyze changes in salivary and systemic CRP. For comparison, salivary and systemic concentrations of inflammatory cytokine interleukin-6 (IL-6) were assessed.

## Materials and methods

### Study population

Nineteen Caucasian patients newly scheduled for anti-TNFα therapy in Poznan University of Medical Sciences were included. All patients fulfilled criteria for the diagnosis of rheumatoid arthritis (RA) according to the American-European Consensus Group classification criteria (Aletaha et al. 2010) (*n* = 10) or the modified New York criteria for ankylosing spondylitis (AS) (van der Linden et al. 1984) (*n* = 9). All patients had an active disease (DAS-28 > 5.1 or BASDAI > 4) and received anti-TNFα treatment according to the standard European League Against Rheumatism (EULAR) recommendations (Braun et al. 2011; Smolen et al. 2014). None of the patients suffered from a coexisting Sjögren’s syndrome. All procedures performed in studies involving human participants were in accordance with the ethical standards of the institutional and/or national research committee and with the 1964 Helsinki Declaration and its later amendments or comparable ethical standards. The study was approved by the Poznan University of Medical Sciences Bioethics Committee (No. 1067/15) and all patients gave their informed consent. The patients were evaluated before and after 12 weeks of therapy.

### Treatment

Patients received anti-TNFα treatment (adalimumab, certolizumab, golimumab, or infliximab) as per standard protocols (Braun et al. 2011; Smolen et al. 2014). All patients had previously been treated with (at least) two synthetic disease-modifying anti-rheumatic drugs (DMARDs) (methotrexate, leflunomide, sulfasalazine, or cyclosporine) (for RA) or non-steroid anti-inflammatory drugs (NSAIDs) (for AS) with no satisfactory effects.

### Assessment of disease activity

Disease activity for RA was assessed by the Modified Disease Activity Scores (DAS)—that include different 28-joint counts and erythrocyte sedimentation rate (DAS28_ESR_) (Prevoo et al. 1995). The therapeutic response in RA patients was defined by the EULAR criteria (van Gestel et al. 1996). The response to therapy in patients with AS was defined as a reduction of ≥ 50% in Bath Ankylosing Spondylitis Disease Activity Index (BASDAI) (Brandt et al. 2000; Garrett et al. 1994).

### Oral health parameters

The patients were asked to answer a questionnaire regarding oral hygiene habits. Clinical examination was performed according to standard practices and parameters of dental (DMFT and DMFS), gingival (GI and SBI), and periodontal (PD and CAL) status as well as of oral hygiene status (API and PLI) were assessed by routine methods (Benamghar et al. 1982; Lange et al. 1977; Loe and Silness 1963).

### Laboratory analysis

Unstimulated whole mixed saliva was collected using the Salivette^®^ (Sarstedt Laboratories; Germany) system, with a sponge gently chewed for 5 min (Poll et al. 2007). Samples of serum were collected by routine methods. All samples were taken at the time of clinical examination in a fasting state and then aliquoted and stored at − 80 °C until assayed. Salivary and serum concentrations of CRP and IL-6 were measured with high-sensitivity ELISA immunoassays (from BioVendor LM; Czech Republic and Diaclone; France, respectively), as per manufacturers’ instructions. All other laboratory tests were performed routinely by the hospital central laboratory.

### Statistical analysis

Statistical analysis was performed using STATISTICA 10.0 software (StatSoft Polska, Krakow, Poland). The data are presented as medians and interquartile ranges or as percentages, as appropriate. As the data obtained did not consistently display a normal distribution (as assessed by the Shapiro–Wilk test), they were analyzed with nonparametric statistics using the Wilcoxon test for paired data. Categorized data were analyzed with the chi-square test. Correlations between variables were analyzed with the Spearman’s rank correlation coefficient. Differences were considered significant at *p* < 0.05.

## Results

The patients’ baseline characteristics are summarized in Table 1. 12 weeks of biologic treatment resulted in a significant improvement in the majority of the patients, with only one patient identified as a non-responder according to EULAR. The favorable response to therapy was reflected by both clinical and biochemical criteria (Table 2). It was also associated with a significant decrease in serum CRP and IL-6 (Table 2).

**Table 1:** Patients’ baseline characteristics (*n* = 19)

Demographic and clinical features	
Age (years)	46 (36–61)
Men (%)	10 (53%)
Disease duration (years)	6 (3–12)
Current smoking (%)	1 (5%)
Oral health parameters	
No/mild periodontitis (%)	8 (42%)
Severe periodontitis (%)	11 (58%)
Plaque Index (PLI)	0.7 (0.4–1.0)
Approximal Plaque Index (API) (%)	75.0 (42.9–100.0)
Sulcus Bleeding Index (SBI)	0.0 (0.0–0.3)
Gingival Index (GI)	0.4 (0.0–1.0)
Probing pocket depth (PD) (mm)	0.8 (0.6–1.3)
Clinical attachment level (CAL)	1.4 (0.6–2.0)
DMFT Index	18.5 (15.0–26.0)

Data presented as medians (and interquartile ranges) or %

**Table 2:** Selected parameters before and after treatment

	Before treatment (*n* = 19)	After 12 weeks of treatment (*n* = 19)	*p* value (Wilcoxon test)
DAS28_(ESR)_ (for RA; *n* = 10)	6.2 (5.5–6.4)	3.5 (2.8–4.5)	**0.005**
BASDAI (for AS; *n* = 9)	7.9 (6.6–8.6)	2.8 (2.0–4.2)	**0.008**
ESR (mm/h)	30 (8–70)	6 (4–24)	**0.002**
WBC (10^3^/l)	9.3 (8.2–9.9)	8.0 (6.4–9.7)	**0.015**
N/L ratio	3.0 (2.4–3.6)	1.4 (1.2–2.1)	**<0.001**
Serum CRP (mg/l)	10.24 (4.65–24.31)	1.52 (0.54–4.13)	**0.010**
Serum IL-6 (pg/ml)	14.23 (5.03–34.61)	2.32 (1.49–25.14)	**0.044**
Salivary CRP (mg/l)	0.30 (0.02–3.72)	0.05 (0.00–1.87)	0.098
Salivary IL-6 (pg/ml)	1.91 (0.94–2.43)	1.48 (0.98–2.78)	0.811

Bold values are statistically significant (*p* < 0.05)

Data presented as the median (interquartile range)

DAS28, 28-joint disease activity score; BASDAI, bath ankylosing spondylitis disease activity Index; ESR, erythrocyte sedimentation rate; CRP, C-reactive protein; N/L ratio, neutrophils to lymphocytes ratio; IL-6, interleukin-6; RA, rheumatoid arthritis; AS, ankylosing spondylitis

**Fig. 1:**
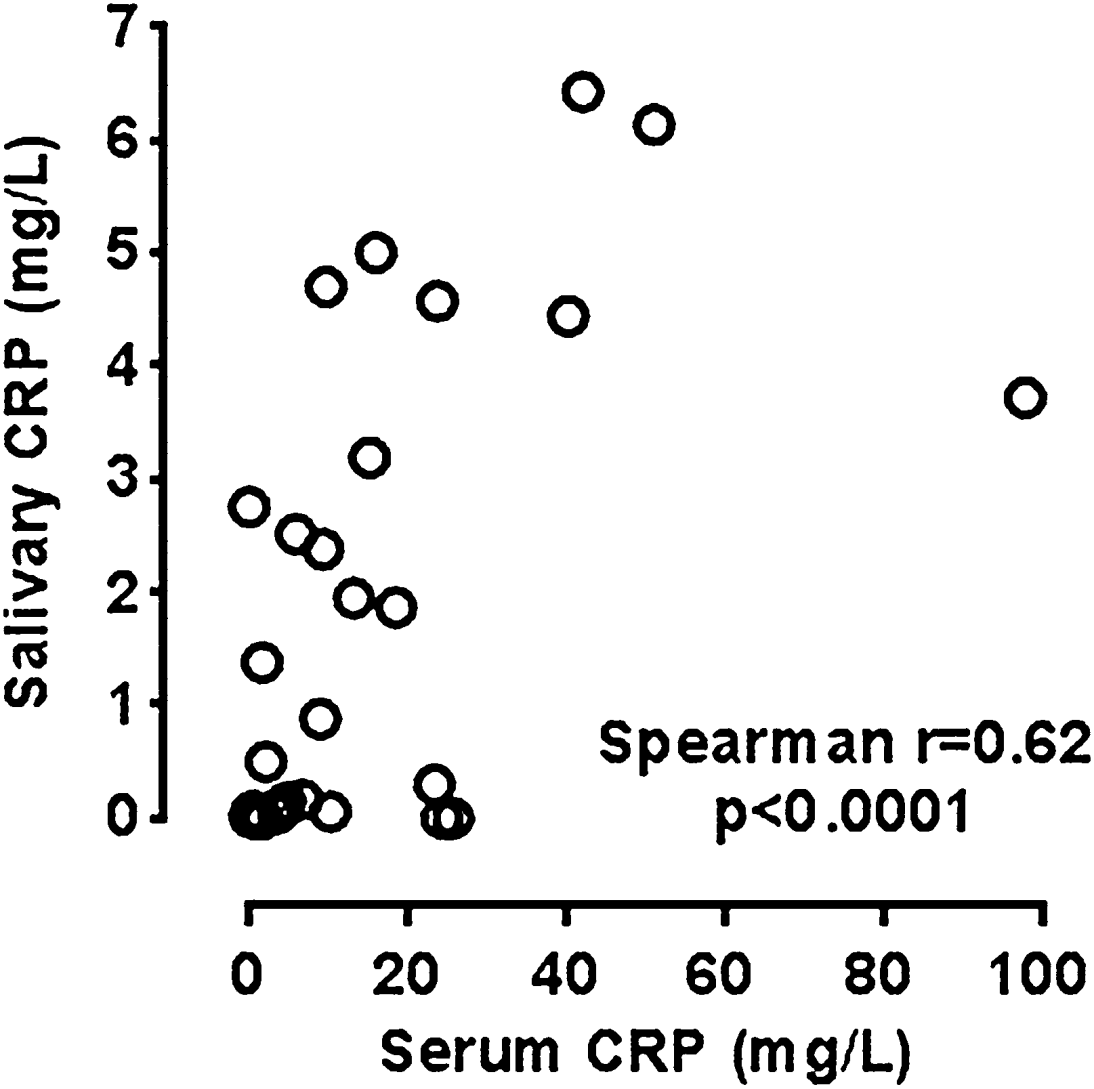
Correlation between serum and salivary CRP

Concentrations of CRP in saliva measured in all patients at all points correlated significantly with those in serum (*R* = 0.62; *p* < 0.0001) (Fig. 1).

**Fig. 2:**
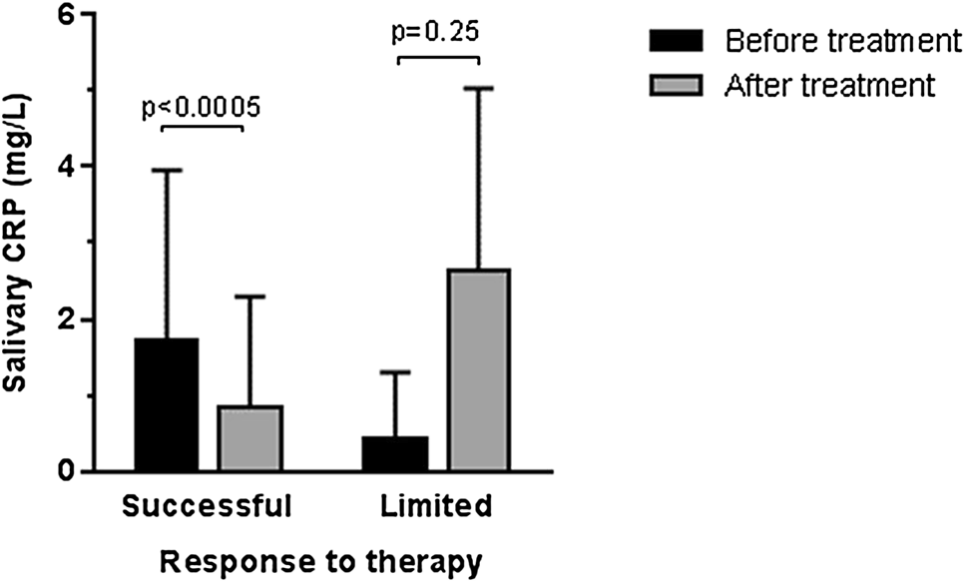
Changes in salivary CRP levels after treatment

Importantly, these correlations were evident independently at the beginning and after treatment (*R* = 0.49; *p* = 0.031 and *R* = 0.63; *p* = 0.004; respectively). The fractional mean changes in serum CRP that occurred as a result of anti-TNFα treatment were reflected by similar percentage changes in CRP levels in saliva (*R* = 0.51; *p* = 0.025). In patients with successful response to treatment significant decrease in salivary CRP levels were observed (*p* = 0.0005) (Fig. 2). In three patients with a limited response to treatment (with a relatively small decrease in clinical disease activity: DAS28 or BASDAI) and with increase of serum CRP levels, an increase in salivary CRP concentrations after treatment was also observed, although it was not statistically significant (*p* = 0.25) (Fig. 2).

Whereas in a single patient defined as an EULAR non-responder, serum CRP concentrations decreased, but still remained high in absolute values (97.96 vs. 42.11 mg/l), salivary CRP levels were both high at baseline and further increased with time (3.72 vs. 6.41 mg/l; NS).

In addition to correlations with serum CRP, salivary CRP correlated with other standard laboratory markers used in RA monitoring [ESR (*R* = 0.60; *p* < 0.001) and N/L ratio (*R* = 0.51; *p* = 0.001)]. There was no consistent association between salivary CRP and oral health parameters.

**Fig. 3:**
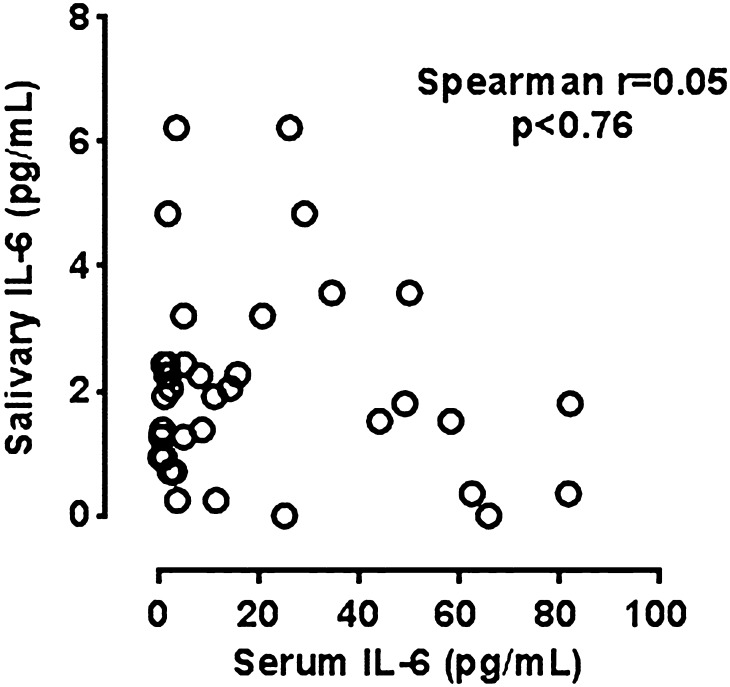
Correlation between salivary and serum IL-6

In contrast to apparent correlation between systemic and salivary CRP, the concentrations of IL-6 in saliva did not correlate with those in serum (Fig. 3), either before or after treatment.

**Fig. 4:**
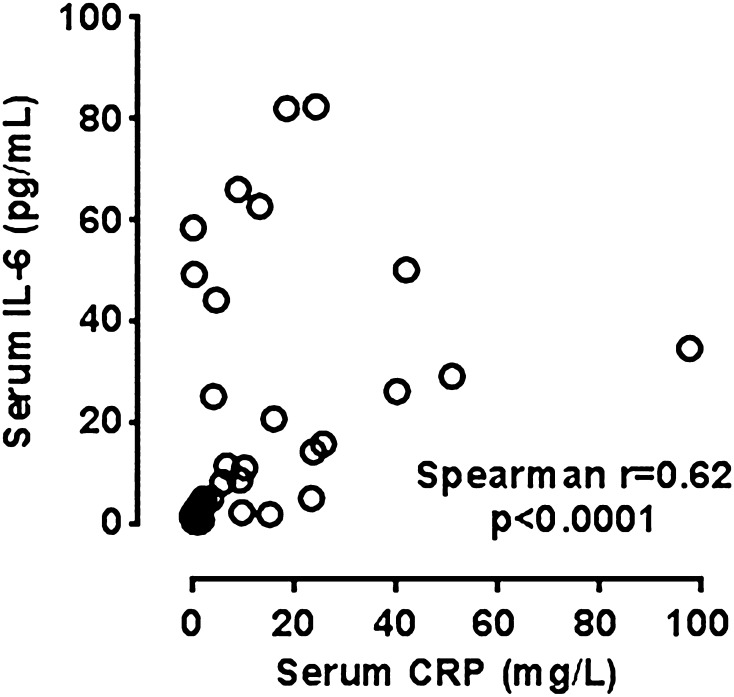
Correlation between serum CRP and serum IL-6

There was also no correlation between salivary CRP and IL-6 levels (both before and after treatment). Interestingly, however, there was still a correlation between serum CRP and serum IL-6 (*R* = 0.62; *p* < 0.001) (Fig. 4).

The salivary concentrations of IL-6 did not change significantly over the course of anti-TNFα treatment and they did not correlate with other systemic inflammatory parameters (ESR, leukocytes, N/L ratio).

## Discussion

The main observation of this exploratory study was that the salivary concentrations of CRP in patients with rheumatic disease correlated significantly with those in serum and paralleled changes in the disease activity as reflected both by clinical and standard biochemical criteria.

To the best of our knowledge, it is the first assessment of how well changes in the salivary CRP concentrations reflect the course of a rheumatic disease. While few earlier studies have reported on the potential use of salivary CRP as an indicator of systemic inflammation (Abdul Rehman et al. 2017; Pallos et al. 2015), especially in neonatology (where non-invasive collection of diagnostic material is of particular importance) (Iyengar et al. 2014; Omran et al. 2017a, b).In this respect, Iyengar et al. demonstrated that salivary CRP is a good index of clinically relevant serum CRP thresholds in neonates (Iyengar et al. 2014). Similar results were obtained also for adults with chronic diseases, including cardiovascular (Labat et al. 2013; Out et al. 2012) and renal disease (Pallos et al. 2015). Our data indicate that salivary CRP could also reflect the activity of rheumatic disease.

Surprisingly, there was no consistent association between salivary CRP and oral health parameters. In this regard, previous studies produced unequivocal results with both the absence (Redman et al. 2016) (Torumtay et al. 2016) and the presence (Nethravathy et al. 2014; Shojaee et al. 2013) of associations of between salivary CRP and the periodontal status. It is possible that an intense systemic inflammatory response in rheumatic disease overshadows that resulting from local lesions in the oral cavity.

We did not observe significant changes in salivary IL-6 over the course of anti-TNFα treatment and we found no correlation of salivary IL-6 with serum levels of either IL-6 or other inflammatory parameters (CRP, ESR, leukocytes, N/L ratio). Other studies detected only a weak correlation between salivary and serum IL-6 levels (Dekker et al. 2017; Slavish et al. 2015). At the same time, patients with RA were reported to have a tendency for higher levels of IL-6 in saliva (Silvestre-Rangil et al. 2017). The main source of the increase levels of CRP in saliva could be crevicular fluid, due to the greater incidence of periodontal disease observed in these patients (Rajkarnikar et al. 2013). Although in our study there was no consistent correlation between salivary IL-6 and periodontal parameters, which may result from a small study group.

An obvious limitation of our study is the small and heterogeneous group of patients analyzed. Thus, it should be viewed as preliminary and be validated in an independent and larger more homogeneous patients’ population.

## Conclusions

In conclusion, salivary concentrations of CRP, but not of IL-6, in patients with rheumatic disease significantly decrease over the course of successful anti-TNFα therapy and parallel changes in the disease activity. In addition, they reflect serum concentrations of both CRP and other surrogate inflammatory markers. Therefore, the measurement of salivary CRP could be of potential use for the assessment of the rheumatic disease activity.
